# Chronic Iron Deficiency as an Emerging Risk Factor for Osteoporosis: A Hypothesis

**DOI:** 10.3390/nu7042324

**Published:** 2015-04-02

**Authors:** Laura Toxqui, M. Pilar Vaquero

**Affiliations:** Department of Metabolism and Nutrition, Institute of Food Science, Technology and Nutrition (ICTAN), Spanish National Research Council (CSIC), C/José Antonio Novais 10, Madrid 28040, Spain; E-Mail: laura.toxqui@ictan.csic.es

**Keywords:** iron deficiency, anemia, iron overload, bone remodeling, bone health, osteoporosis, vitamin D, bone formation, bone resorption

## Abstract

Iron is essential in oxygen transport and participates in many enzymatic systems in the body, with important roles in collagen synthesis and vitamin D metabolism. The relationship between iron and bone health comes from clinical observations in iron overload patients who suffered bone loss. The opposite scenario—whether iron deficiency, with or without anemia, affects bone metabolism—has not been fully addressed. This is of great interest, as this nutrient deficiency is a worldwide public health problem and at the same time osteoporosis and bone alterations are highly prevalent. This review presents current knowledge on nutritional iron deficiency and bone remodeling, the biomarkers to evaluate iron status and bone formation and resorption, and the link between iron and bone metabolism. Finally, it is hypothesized that chronic iron deficiency induces bone resorption and risk of osteoporosis, thus complete recovery from anemia and its prevention should be promoted in order to improve quality of life including bone health. Several mechanisms are suggested; hence, further investigation on the possible impact of chronic iron deficiency on the development of osteoporosis is needed.

## 1. Iron in Human Health

Iron is the fourth most common element on earth and is a biologically essential component of every living organism [1]. The body requires iron for oxygen transport, in particular by the proteins hemoglobin and myoglobin, and for the formation of heme enzymes and other iron-containing enzymes involved in electron transfer and oxidation-reduction reactions [2]. Approximately 73% of the body’s iron is in the hemoglobin of circulating red cells and in the muscle protein myoglobin; 12% is in iron storage proteins, and another 15% is critically important in dozens of enzymes that are essential for the functioning of all cells and tissues [3].

Iron deficiency is so widespread in humans that it affects all ethnic groups, in both industrialized and non-industrialized countries, with major consequences for human health and socioeconomic development [1,2,3,4]. A low iron status can reduce physical activity and increase susceptibility to infections. Moreover, iron deficiency, even in the absence of anemia, can cause fatigue and reduce work performance [5]. It has been observed in young women that a good iron status enhances various components of wellbeing [6].

### 1.1. Prevalence and Risk Factors of Iron Deficiency Anemia

The World Health Organization (WHO) defines anemia as a condition in which the number of red blood cells or their oxygen-carrying capacity is insufficient to meet physiological needs, which vary by age, sex, altitude, smoking, and pregnancy status. Iron deficiency anemia is the most common form of anemia [4].

Globally, iron deficiency anemia affects between 1.5 and 1.7 billion people, which is 24.8% of the world population. Even though the highest prevalence is in preschool-age children (47.4%, 283–303 million children), the population group with the greatest number of individuals affected is women of childbearing age (30.2%, 446–491 million women) [4].

Iron deficiency results when iron losses or requirements exceed absorption, and is often multifactorial. It is well established that low iron consumption and low bioavailability of dietary iron are crucial in the development of iron deficiency [5]. The bioavailability of non-heme iron, the main source of iron from most diets (approximately 85%–90% of total iron intake), is affected by the presence of several dietary components. Non-heme iron absorption is enhanced principally by animal tissue (especially red meat) and ascorbic acid [7,8], whereas phytic acid, calcium, and polyphenols are the main inhibitors [7,9,10,11,12].

Menstrual blood loss contributes to a negative iron balance in women of childbearing age [13,14]. Therefore, these women constitute an important risk group due to the additional iron demands of menstruation and pregnancy [13,14,15]. Other factors in postmenopausal woman and men are malabsorption (*i.e.*, celiac disease), ulcerative colitis, use of nonsteroidal anti-inflammatory drugs, or gastrointestinal blood loss [16].

In addition, genetic factors are determinant in the development of iron deficiency anemia. Preliminary absorption assays and genome-wide association studies identified genetic variants that potentially could be genetic markers of iron deficiency [17,18]. Blanco-Rojo *et al*. (2011) [19] observed in Spanish menstruating women that a large percentage of the genetic variation of serum transferrin was explained by two single nucleotide polymorphisms (SNPs) located in the transferrin (Tf) gene and two in the hemochromatosis (HFE) gene. Only one of them, located in an intronic region of the Tf gene (SNP rs3811647), was associated with iron deficiency and affected transcription [20], while the others appear to have compensating effects, particularly the very well-known Cys282Tyr substitution in HFE, suggesting that this genetic factor that is characteristic of iron overload protects from iron deficiency anemia. Other genetic variants, such us SNP rs1375515 located in a calcium channel gene, are also related to hematological iron markers and clinical phenotypes [21]. The study of the influence of dietary, menstruation, and genetic factors combined reveals that meat, menstrual blood loss, and the C282Y genetic variant determine iron status in young women [13].

### 1.2. Functional Consequences of Iron Deficiency

Iron deficiency, even in absence of anemia, adversely affects:

- The cognitive performance and physical growth of infants, preschool-, and school-aged children [22]. Among the cognitive impairments, those referring to attention span, intelligence, and sensory perception functions are well known, as well as those associated with emotions and behavior [23].
- The immune status and morbidity from infections of all age groups [24].
- The use of energy sources by muscles and thus the physical capacity, motor skills, work, and athletic performance of adolescents and adults of all age groups [22,23,24,25].
- During pregnancy, iron deficiency anemia increases perinatal risks for mothers and neonates and increases overall infant mortality [22].

### 1.3. Evaluation of Iron Status

**Figure 1:**
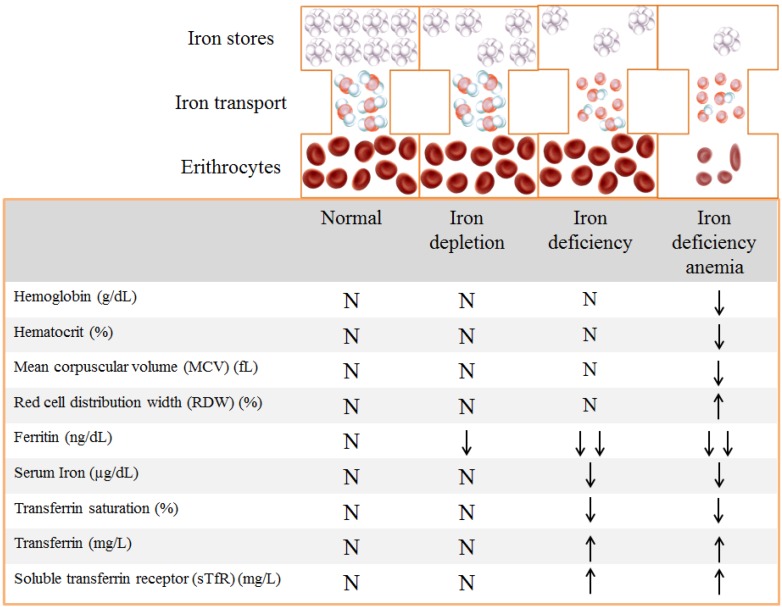
Biochemical markers of iron status [10].

Iron status is characterized by three phases: iron depletion, iron deficiency (iron deficient erythropoiesis or non-anemic iron deficiency), and iron deficiency anemia [10,26]. The terminology and cut-off values for the biochemical measures of each of these stages vary as some functional changes may occur in the absence of anemia (Figure 1).

In the first phase (iron depletion), body iron stores are depleted, which can be detected by a reduction in serum ferritin. The second phase (iron deficiency) is characterized by the reduction in serum ferritin and serum iron, and an increase in serum transferrin and its receptor (sTfR) in an attempt to enhance iron transport to tissues, but transferrin saturation falls. At this stage some iron-dependent functions, like erythropoiesis, are compromised. In the final phase (iron deficiency anemia), oxygen supply to tissues is impaired, which is reflected by a decrease in hemoglobin concentrations, hematocrit, mean corpuscular volume (MCV), mean corpuscular hemoglobin (MCH), serum ferritin, serum iron, and transferrin saturation [10].

Table 1 shows the hemoglobin cut-off values to diagnose anemia [22].

**Table 1:** Hemoglobin cut-off levels (g/L) to diagnose anemia.

Population	Non-Anemia	Anemia		
		Mild	Moderate	Severe
Children 6–59 months	>110	100–109	70–99	<70
Children 5–11 years	>115	110–114	80–109	<80
Children 12–14 years	>120	110–119	80–109	<80
Non-pregnant women (>15 years of age)	>120	110–119	80–109	<80
Pregnant women	>110	100–109	70–99	<70
Men (>15 years of age)	>130	110–129	80–109	<80

Iron balance is regulated by absorption in the enterocytes, but once absorbed there is no metabolic route for excretion [27] and the major amount of iron entering blood plasma comes from recycling. Iron can form free radicals, and excess iron in body tissues can lead to tissue damage [28]. Only small amounts of iron are lost through cell shedding and minor bleeding, and these are replaced by the absorption of similar amounts by duodenal enterocytes [29]. The main regulator of iron metabolism is hepcidin. This hormone inhibits iron absorption, decreases release of iron from macrophages, and releases stored iron from hepatocytes. Hepcidin is regulated by three pathways: lower iron stores, erythropoietic signals, and inflammation. Body iron deficiency induces low hepcidin synthesis and, consequently, iron movement into plasma is enhanced [28,30].

## 2. Bone Metabolism

Bone is an active and dynamic connective tissue, formed by cells and the extracellular matrix, which continually remodels itself to adapt to the influences of growth and changes in mechanical loads, to maintain mineral homoeostasis, and to regulate the bone marrow environment. Bone tissue is largely composed of type I collagen and the remaining organic components consist of non-structural proteins like osteonectin, osteocalcin, fibronectin, *etc*. The inorganic phase of bone is mainly composed of calcium and phosphate (hydroxyapatite, Ca_10_(PO_4_)_6_(OH)_2_) [31].

### 2.1. Bone Remodeling

During development and growth, the skeleton is sculpted to achieve its shape and size by the removal of bone from one site and deposition at a different one; this process is called modeling [32,33]. Once the skeleton has reached maturity, regeneration continues in order to maintain skeletal integrity by removing the foci of damaged bone and replacing them with new bone [33]; this process is called remodeling and is responsible for the complete regeneration of the adult skeleton every 10 years [32]. Bone remodeling occurs through the concerted action of a functional cohort of cells called the basic multicellular unit (BMU). The BMU consists of the osteoclasts resorbing bone, the osteoblasts replacing bone, the osteocytes within the bone matrix, the bone lining cells covering the bone surface, and the capillary blood supply [32,34]. Bone remodeling occurs in 4 phases: activation, resorption, reversal, and formation [34,35].

**Figure 2:**
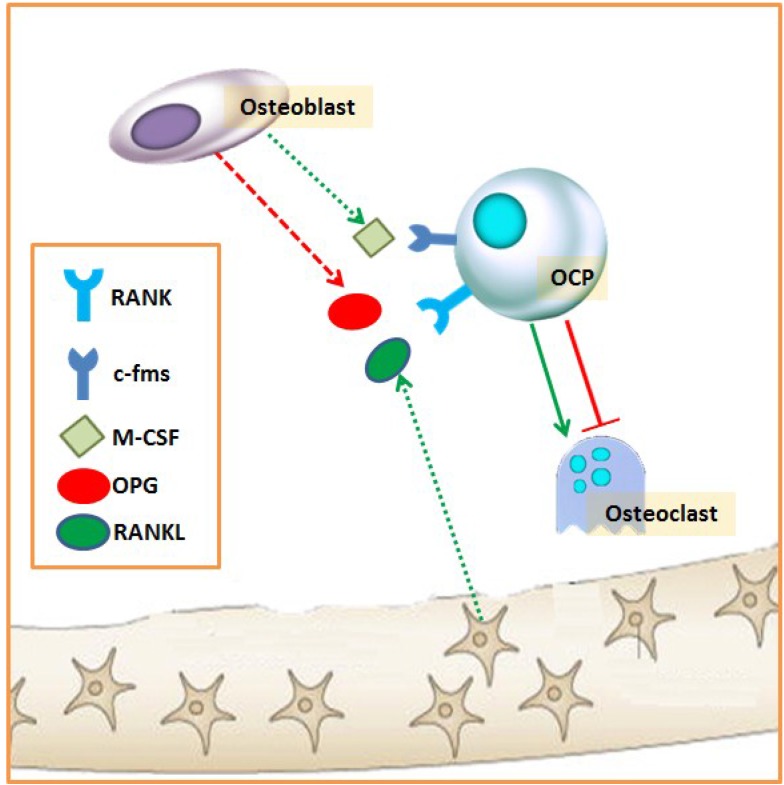
Activation phase of bone remodeling. c-fms: M-CSF receptor; M-CSF: macrophage colony-stimulating factors; OCP: osteoclast precursor; OPG: osteoprotegerin; RANKL: receptor activator of NF-kB ligand; RANK: RANKL receptor.

The activation phase consists of the recruitment of osteoclast precursors (OCP) (from hematopoietic stem cells), their differentiation into mature osteoclasts, and their activation (Figure 2) [34,35]. Osteoclast activity and formation are dependent on the presence of two different cytokines, which are essential and sufficient: macrophage colony-stimulating factors (M-CSF) and receptor activator of NF-κB ligand (RANKL). RANKL is expressed by several cell types in bone and bone marrow, including osteoblasts, osteocytes, bone marrow stromal cells, and lymphocytes. Osteoblastic cells have been thought to be the major cell type that expresses RANKL; however, Nakashima *et al*. [36] found that purified osteocytes express a much higher amount of RANKL and have a greater capacity to support osteoclastogenesis *in vitro* than osteoblasts and bone marrow stromal cells. Osteocytes are cells located within the bone matrix and are former osteoblasts that become surrounded by unmineralized matrix (osteoid) during bone formation. These cells constitute around 90%–95% of the adult bone cell population [37,38] and are now recognized as the major regulator of skeletal activity, capable of sensing and integrating mechanical and chemical signals from their environment to regulate both bone formation and resorption [37]. The receptor of RANKL in OCP is RANK, which initiates events that result in osteoclast differentiation. In contrast, the decoy receptor of RANK, the osteoprotegerin (OPG) produced by the osteoblasts, has an essential physiological role as an inhibitor of osteoclast formation [39,40]. Another factor required for progenitor cells to differentiate into osteoclasts is M-CSF, which binds to c-fms in OCP [41].

Osteoclasts adhere to bone and resorb it by acidification and proteolysis of the bone matrix. In the further course of resorption, the osteoclast progenitors are differentiated to fully active osteoclasts that dig trenches with a depth of 40–60 μm in the bone. Osteoclast function is regulated both by locally and systemic factors: calcitonin, androgens, thyroid hormone, insulin, parathyroid hormone (PTH), insulin-like growth factor (IGF)-I, Platelet-derived growth factor (PDGF), inflammation, acidosis, and hypoxia [42].

The resorption phase is followed by the reversal phase, which is a transition from osteoclast to osteoblast activity and comprises the differentiation of osteoblast precursors and the discontinuation of bone resorption with osteoclast apoptosis [31]. During this phase, bone-resorbing osteoclasts stimulate differentiation of osteoblast precursors, activating bone formation in bone resorption lacunae. In that sense, bone resorption may liberate growth factors such as TGF-b, bone morphogenetic proteins (BMPs), and insulin-like growth factor (IGF)-II from bone matrix, which in turn activate bone formation [43,44].

Several factors stimulate osteoclast apoptosis, including high extracellular calcium concentrations that result from bone degradation [45], estrogens, and pharmacologic doses of bisphosphonates [33].

After osteoblast activation, osteoblasts lay down new bone material (*i.e.*, collagen type I, osteocalcin, osteopontin, *etc*.) until the resorbed bone is entirely replaced by a new one [31].

The osteoblast represents a unique bone-forming cell derived from mesenchymal stem cells. These cells move in to cover the excavated area and begin the process of new bone formation by secreting osteoid, which is eventually mineralized into new bone. This stage lasts the longest, as bone formation is slower than bone resorption [34,35]. Finally, the osteoblasts become quiescent at the end of bone remodeling, and they form flattened lining cells on the bone surface until a new remodeling cycle is triggered or become osteocyte cells (as reviewed previously, osteocytes are cells derived from osteoblasts embedded in bone) [31].

### 2.2. Biochemical Markers of Bone Remodeling

Biochemical bone turnover markers are released during bone remodeling and provide a measure of the rate of bone metabolism. They comprise enzymes secreted by osteoblasts and osteoclasts during remodeling, degradation products formed during resorption, and precursors released during new bone formation. They reflect metabolic abnormalities such as accelerated bone turnover (Table 2) [46].

**Table 2:** Biochemical bone turnover markers in serum and urine.

Bone Resorption Marker	Bone Formation Marker
C-terminal cross-linked telopeptide of type I collagen (CTx)	Bone alkaline phosphatase (ALP)
N-telopeptide cross-linked of type 1 collagen (NTx)	Osteocalcin (OC)
Pyridinoline (PYD)	N-terminal propeptide of type I procollagen (P1NP)
Deoxypyridinoline (DPD)	C-terminal propeptide of type I procollagen (P1CP)
Hydroxyproline (HYP)	
Tartrat-resistant acid phosphatase type 5b (TRAP 5b)	

## 3. Role of Iron in Bone Metabolism

**Figure 3:**
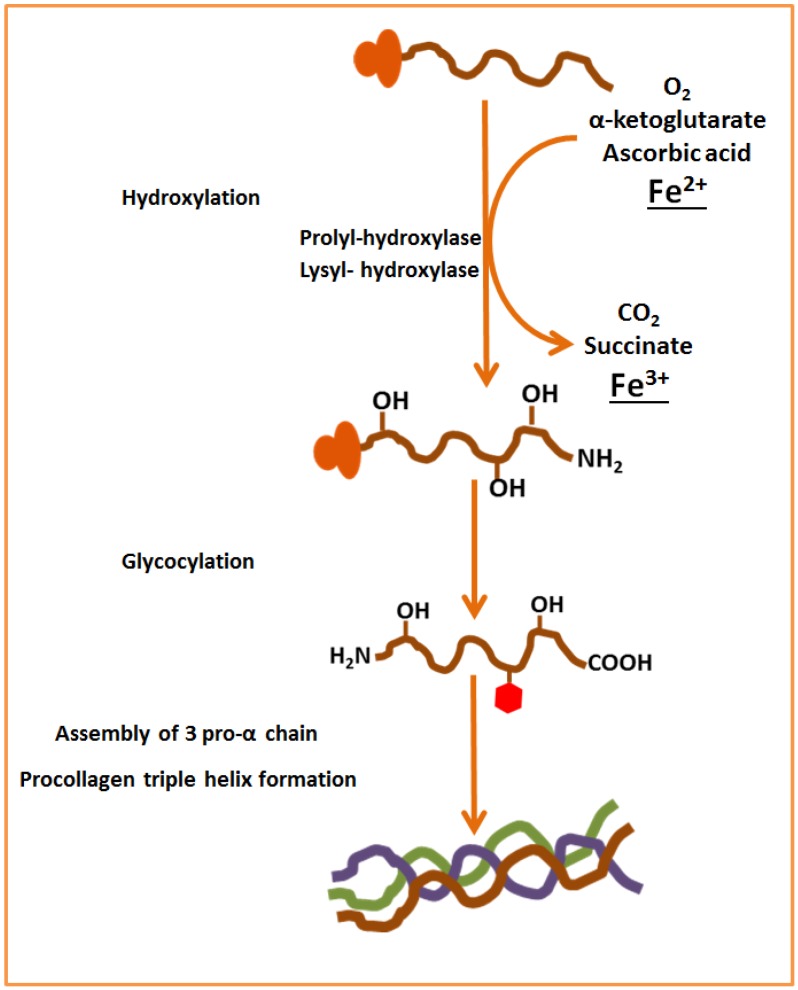
Role of iron in the collagen synthesis.

Iron participates in a variety of enzymatic systems in the body, including the enzymes involved in collagen synthesis. Collagen is the most abundant protein in animals, and the major component of connective tissue [47]. Regarding bone tissue, about 90% of total bone protein is composed of type I collagen [31]. For collagen synthesis, first, a three-dimensional stranded structure is assembled, with the amino acids glycine and proline as its principal components. This is not yet collagen but its precursor, procollagen. Procollagen is then modified by the addition of hydroxyl groups to the amino acids proline and lysine. This step is important for later glycosylation and the formation of the triple helix structure of collagen. This reaction requires α-ketoglutarate, molecular oxygen, ferrous iron, and a reducing agent [48,49]. In this regard, ascorbate reduces the inactive Fe^3+^ state to the active Fe^2+^ state [50]. During the reaction, α-ketoglutarate is decarboxylated oxidatively to produce succinate and CO_2_ [48] (Figure 3). These hydroxylation reactions are catalyzed by two different enzymes: prolyl-4-hydroxylase [48] and lysyl-hydroxylase [49].

Another mechanism in which iron participates in bone metabolism is through vitamin D activation and deactivation. In this pathway, the cytochrome P450 superfamily, a large number of heme-containing monooxygenases, plays an important role [51].

Vitamin D undergoes two steps of hydroxylation for its activation. The first step occurs in the liver and as a result 25-hydroxyvitamin D (25OHD) is produced. This is the first and bounden step in the production of the active form of this vitamin [51,52]. This step occurs in the liver and is catalyzed by the cythocrome P-450 25-hydroxylase (CYP2R1) [51]. Mutations in CYP2R1 gene lead to low serum levels of 25OHD and are associated with rickets (softening and weakening of bones in children, leading to bone deformities and fractures). CYP2R1 deficiency is designated as vitamin D-dependent rickets type 1B [53].

The second step occurs in the kidney, producing 1,25-dihydroxyvitamin D (1,25OHD), the bioactive form of the vitamin D, which binds to the vitamin D receptor and regulates calcium metabolism. Thus this second hydroxylation regulates the level of biologically active vitamin D and plays an important role in calcium homeostasis. Active 1,25OHD promotes intestinal calcium and phosphorous absorption, phosphate reabsorption in the kidney, and calcium and phosphate release from the bone [52,54,55]. The enzyme responsible for this reaction is the 25-hydroxyvitamin D 1α-hydroxylase (CYP27B1). Inactivating mutations in CYP27B1 cause vitamin D-dependent rickets type 1A, a rare autosomal recessive disorder characterized by the early onset and severe syndrome of rickets [56].

**Figure 4:**
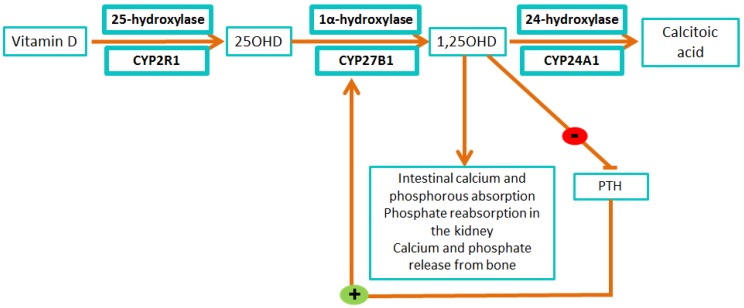
Vitamin D activation and deactivation by the cytochromes P450.

Finally, the 1α,25-hydroxyvitamin D 24-hydroxylase (CYP24A1), inactivates the 1,25OHD through multiple oxidations of the sterol side chain (Figure 4). Loss of CYP24A1 function usually leads to idiopathic infantile hypercalcemia, a rare disease characterized by failure to thrive, vomiting, dehydration, and nephrocalcinosis [57].

Iron is essential for vitamin D metabolism, as all of the vitamin D-related cytochromes catalyze single or multiple hydroxylation reactions on specific carbons of the vitamin D substrate using a heme-bound iron [58].

## 4. Relationship between Body Iron Levels and Bone Metabolism

A relationship between iron metabolism and bone was first established from clinical studies that observed a higher incidence of osteoporosis and fractures in patients with disorders of iron metabolism such as hereditary hemochromatosis, thalassemia, and sickle cell disease [59,60,61].

In healthy populations the relationship between iron status and bone metabolism is controversial. Results show positive correlation between serum ferritin and BMD in elderly men but not in women [62]. In contrast, there was a negative correlation in women older than 45 years of age [63], and no association between BMD and either transferrin saturation or ferritin in men [64]. Buyukbese *et al*. [65] did not observe differences in ferritin levels between osteoporotic postmenopausal women and controls. The following sections describe the role of iron overload and iron deficiency on bone metabolism and the possible mechanisms involved.

### 4.1. Iron Overload and Bone Loss

Thalassemia refers to a group of inherited disorders of hemoglobin synthesis owing to a defect in the ability of erythroblasts to synthesize one or more of the globin chains of hemoglobin. In its more severe form (β thalassemia major and α thalassemia major), ineffective erythropoiesis and the subsequent hemolytic anemia require chronic transfusion. In this condition, iron overload occurs as a complication of chronic transfusion therapy.

Several bone abnormalities are present in patients with thalassemia major, including the enlargement of the head bones, spinal deformities, scoliosis, nerve compression, bone loss, and fractures. Frequently they suffer from bone pain. The pathogenesis of osteoporosis is very different form that of bone deformity [66]. The reported frequency of osteoporosis, in thalassemia mayor patients, despite adequate transfusion and iron chelation therapy, varies from 13.6% to 50% with an additional 45% affected by osteopenia [67]. Multiple factors are involved in the pathogenesis of osteopenia or osteoporosis in thalassemia major, such as: delay in sexual maturation, hypothyroidism, parathyroid gland dysfunction, accelerated hemopoiesis with progressive bone marrow expansion, diabetes, and low secretion of growth hormone or IGF-I [66]. Although endocrine dysfunction has a major role in the development of osteoporosis in thalassemia mayor, iron overload due to transfusion and chelation therapy also influence bone [68].

Genetic hemochromatosis is a hereditary disease characterized by a mutation in the HFE gene, C282Y, or H63D, and excessive iron absorption particularly in C282Y homozygous. The prevalence of osteoporosis in hemochromatosis has been estimated at between 25.3% and 34.2% [69]. One study carried out in men with hemochromatosis observed osteopenia in 78.9% of patients and osteoporosis in 34.2%, and that femoral neck BMD appeared to fall with rising hepatic iron concentrations [61]. In addition, untreated hemochromatosis may lead to various joint complications including arthritis and dermoskeleton alterations [64].

Sickle cell anemia is another genetic disorder in which sickle hemoglobin leads to tissue hypoxia and adverse effects on bone. Patients experience acute episodes (vaso-occlusive bone or joint pain, or acute osteomyelitis) as well as chronic bone and joint disorders. Combined, osteopenia and osteoporosis have been found in up to 80% of adults with sickle cell disease anemia, but their association with fracture risk has not been studied in depth [70,71,72]. Bone loss has been ascribed in part to bone marrow hyperplasia and inflammation secondary to chronic anemia or bone marrow ischemia [71,73,74]. Table 3 summarizes the effects of iron overload on bone health in humans.

**Table 3:** Effects of iron overload on bone health in humans.

Subjects		Main Results	Ref.
Hemochromatosis	Men, *n* = 38 (13% hypogonadal)	79% osteopenic; 34% osteoporotic	[61]
Hemochromatosis	Adults, *n* = 87	41% osteopenic; 25% osteoporotic, Lower BMD and decrease in bone formation in patients with higher tissue iron	[75]
Thalassemia	Adults, *n* = 80. BMD	Negative association between ferritin levels and BMD	[76]
Thalassemia	All, *n* = 702; *n* = 312, BMD	Thalassemia major 17% fracture; thalassemia intermedia 12%	[77]
Thalassemia	Adults, *n* = 41	41% osteoporosis	[78]
Thalassemia	Children, *n* = 18	61% low bone mass	[79]
Sickle cell anemia	Adults, *n* = 17	47% osteopenia; hepatic iron and serum ferritin higher in osteopenic than non-osteopenic patients	[80]

The main mechanism by which excess iron is harmful to bone is oxidative stress. Tsay *et al.* [81] developed a mouse model of iron overload in which mice were injected chronically with iron dextran in two doses, resulting in severe tissue iron overload. Iron loading induced changes in bone composition, trabecular and cortical thinning of bone, and bone resorption. Reactive oxygen species ROS and the pro-osteoclatogenic cytokines TNF-α and IL-6 increased in a manner correlated with the severity of iron overload. Interestingly, treatment with the antioxidant *N*-acetyl-l-cysteine prevented the development of bone abnormalities. In view of these findings, the authors conclude that iron excess leads to increased oxidative stress, which induces inflammatory changes that then mediate bone loss through changes in bone resorption.

Another suggested mechanism by which iron overload may interfere in osteoid maturation and mineralization includes the incorporation of iron into crystals of calcium hydroxyapatite, which consequently affects the growth of hydroxyapatite crystals and reduces the BMU tensile strength [68,82]. In addition, iron itself may have an effect on osteoblastic activity, as demonstrated *in vitro* [59] and in hemochromatosis patients who showed lower markers of bone formation [69]. Finally, iron chelators such as deferoxamine inhibit osteoblast and fibroblast proliferation, osteoblast precursor differentiation, and collagen formation, and enhance osteoblast apoptosis, especially in patients who receive inappropriately high doses of these chelators [68].

**Table 4:** Effects of iron deficiency on bone health in animals and humans.

Methods	Bone Determinations	Effects of Iron Deficiency on Bone	Ref.
*Animal studies*			
Weanling female rats: Control diet Calcium restricted diet Iron deficient diet Pair-fed to the iron-deficient group	OC, DPD, serum 1,25OHD, BMD, and BMC (total and femur), femur strength	Decreased: BMD, BMC, and femur bone strength No change: DPD, OC, and 1,25OHD	[83]
Weanling male rats: Control diet Iron-deficient diet	OC, CTx, DPD, BMC, and BMD (femur and lumbar vertebra)	Decreased: OC, DPD, BMC, and BMD Increased: CTx	[84]
Weanling male rats: Control diet Iron-free diet Pair-fed to the iron-deficient group	PTH, serum 1,25OHD, IGF-I, OC, BMC, and BMD (femur)	Decreased: OC, DPD, Serum IGF-I, BMC, and BMD Increased: CTx, No change: PTH and IGF-I	[85]
Weanling male rats Control diet Low-Fe diet	P1NP, TRACP 5b, CTx, PTH, 25OHD, and BMC (sternum and femur)	Decreased: P1NP Increased: PTH, TRACP 5b, and CTX No change: 25OHD, Ca, and P content in sternum	[86]
*Human studies*			
Healthy postmenopausal women (*n* = 242; 40–66 years)	Dietary Fe and Ca, BMD	Dietary iron positively associated with BMD	[87]
Healthy postmenopausal women (*n* = 228; 40–65 years)	BMD at different sites	Dietary iron positively associated with BMD only in women using hormone replacement therapy	[88]
Osteoporotic postmenopausal women (*n* = 455; 66 ± 10 years)	BMD, ALP, OC, 25OHD	Negative correlation between transferrin and BMD	[89]
Mild iron deficient women (*n* = 41; 18–35 years)	25OHD, PTH, ALP, NTx	Positive association between 25OHD and transferrin saturation	[90]
Non-anemic women (*n* = 165; 18–35 years)	25OHD, PTH, P1NP, NTx	Negative correlations: ferritin and NTx; transferrin and P1NP	[91]

### 4.2. Iron Deficiency and Bone Loss

Iron is essential for cell growth and functioning, thus it is not surprising that deficiency anemia affects bone metabolism. In this regard, it is not clear which alteration—iron overload or iron deficiency—may have more important health repercussions and a higher impact on public health. Zhao *et al*. [92] performed an interesting experiment in human osteoblast cells to determine the effects of both excessive iron and low iron on osteoblast activity. They concluded that excess iron inhibited osteoblast activity in a concentration-dependent manner; mild iron deficiency promotes osteoblast activity but severe low iron levels inhibited osteogenesis. Table 4 summarizes the effects of iron deficiency on bone health.

Reports from animal studies have shown the relationship between dietary iron restriction and bone health, and found that severe nutritional iron restriction has a significant impact on bone, affecting BMD, bone mineral content (BMC), and femur strength. In several studies decreases in bone formation and/or increases in bone resorption markers were found [83,85,86]. These parameters were recovered after supplying a normal or high-iron diet [93].

In healthy postmenopausal women, a positive association between dietary iron and BMD was found [87]. However, the same authors observed in a one year follow-up study that this association was only observed in women on hormone replacement therapy [88]. In this line, a positive association between ferritin levels and BMD was found in the KNANES study for the whole cohort (men and women 10–80 years) [94] and for men >65 years [62], but the association was the inverse for women ≥45 years [94]. Other studies report higher serum transferrin and lower but not significant ferritin levels in women with osteoporosis compared to controls [89]. Therefore, the hormonal situation plays a role modulating the effects of iron on bone health.

Our research group studied the iron–bone relationship in menstruating women. They constitute an important population group at risk of iron deficiency anemia and additionally they are at the physiological stage of achieving peak bone mass [90,91,95]. As shown in Table 5, significant negative associations between serum transferrin and P1NP, and between ferritin and NTx were obtained, suggesting a relationship between bone turnover and iron status. When the women are divided into three groups: iron deficiency anemia (hemoglobin <12 mg/dL; serum ferritin <30 ng/mL), iron deficiency (hemoglobin >12 mg/dL; serum ferritin <30 ng/mL), and iron sufficiency (hemoglobin >12 mg/dL; serum ferritin >30 ng/mL), NTx was significantly higher in the iron-deficient compared to the iron deficiency anemia and iron-sufficient groups (Table 6), which suggest that bone resorption is enhanced by mild iron deficiency. This result may be related to the work of Zhao *et al*. [92] mentioned above, which shows that mild iron deficiency promotes osteoblast activity while both excess iron and severe iron deficiency inhibit osteoblasts. Therefore, and although the differences between groups in P1NP were not significant, it is possible that osteoblast and osteoclast activation are coupled, resulting in higher bone resorption, which is faster than bone formation and predominates in this subclinical stage of iron deficiency. The underlying mechanisms and consequences of this finding should be further explored.

**Table 5:** Correlations between iron and bone remodeling biomarkers in menstruating women.

	*n*	Transferrin	Ferritin Log
NTx log	220	0.047	−0.237 *
P1NP	193	−0.254 *	0.055

Pearson’s correlation controlled for age and BMI; * *p* ≤ 0.001.

**Table 6:** Bone biomarkers in iron deficiency anemia, iron deficiency, and iron sufficiency groups.

Parameter	Iron Deficiency Anemia (*n* = 42)	Iron Deficiency (*n* = 94)	Iron Sufficiency (*n* = 84)	ANOVA *p*
25OHD (nmol/L)	56.0 ± 25.0	50.7 ± 25.7	55.1 ± 21.0	NS
P1NP (ng/mL)	40.4 ± 15.9	54.6 ± 21.7	50.8 ± 19.9	NS
PTH pg/mL	39.8 ± 14.1	40.5 ± 16.5	38.1 ± 15.4	NS
NTx (nmol BCF/mmol creatinine)	44.5 ± 26.1	64.5 ± 35.2 *	45.3 ± 38.8	0.001

Values are mean ± SD. Differences between groups (ANOVA); * Different from anemic and deficient groups (*p* < 0.01) by *post hoc* Bonferroni test.

We performed specific interventions in these women. Anemic women were treated with ferrous sulfate, and it was found that those who recovered normal hemoglobin levels exhibited a decrease in bone remodeling, as both P1NP and NTx were lower at the end of treatment compared to baseline [95]. Two nutritional interventions were carried out in iron-deficient women using functional foods. One of them studied the effects of consuming an iron-fortified fruit juice compared to placebo. This functional food was very efficacious in improving iron status [96]; however, iron formation and resorption markers did not change during the 16-week intervention period [90]. The other nutritional intervention investigated the effects of an iron- or iron and vitamin D-fortified dairy product on iron and bone metabolism. This product did not provide bioavailable iron and iron status did not improve [11]; the vitamin D fortification reduced both bone formation and resorption in these women, as expected, but the possible effect of iron on bone could not be seen [91].

It is difficult to explain why the bones of anemic women responded to the iron recovery, but no variation in bone remodeling was observed in the iron-deficient women who consumed the iron-fortified fruit juice, or why bones from iron-deficient women treated with vitamin D clearly improved. Therefore, further studies in this line are needed.

## 5. Mechanisms of the Relationship between Iron Deficiency and Bone Loss

Different mechanisms by which iron deficiency affects bone have been suggested. On the one hand, there is the role of iron as an essential cofactor for hydroxylation of prolyl and lysil residues of procollagen, as detailed before (Figure 2). On the other hand, there is its participation in vitamin D metabolism through the cytochromes P450 (Figure 3). A third mechanism to be considered is hypoxia. A state of hypoxia occurs when oxygen supply to tissues is reduced, as occurs in anemia. Hypoxia is a major stimulator of bone resorption, inducing osteoclastogenesis, which is later followed by osteoblastogenesis [97,98]. Interestingly, during normoxia, α-ketoglutarate and both molecular oxygen and iron are needed for the activity of a prolyl hydroxylase domain protein that acts on the hypoxia inducible factor α (HIF-1α) for its degradation, preventing its action. The role of iron in HIF-1α is similar to that involved in the collagen synthesis, as indicated previously (Figure 3). Under hypoxia conditions, HIF-1α is not degraded and translocates to the nucleus where transcription of >100 genes is regulated [48]. Among these genes, erythropoietin (EPO), PDGF, and transferrin are of particular interest in exploring the link between iron deficiency anemia and bone health. In this regard, apart from the erythropoiesis function, several pleiotropic effects of EPO have been recognized. EPO acts directly or indirectly in the remodeling process [98,99,100] and has been reported to induce bone resorption in mice [101].

Finally, metabolic or local acidosis can also induce osteoclast activation and bone loss [97,102]. Acidosis can also result from hypoxia and it is widely known that bone resorption is activated under low pH in order to release phosphates and restore acid-base equilibrium in extracellular fluid. However, recently Okito *et al*. [102] observed that under acidic conditions osteoblasts tend to change their phenotype from osteogenic to osteoclastogenic, demonstrating that both osteoblast and osteoclast activities are altered.

It is important to remember that bone formation is slower than bone resorption, thus a prolonged situation of iron deficiency could result in bone loss and increase risk of osteoporosis, with the concomitant public health impact.

## 6. Hypothesis and Directions for Further Research

Based on current knowledge, we hypothesize that iron deficiency—with or without anemia—negatively affects bone through different mechanisms.

To prove the hypothesis that chronic iron deficiency predisposes patients to bone loss, osteoporosis, and risk of fracture, various investigation lines should be developed as there are many unsolved issues. It is not known to what extent severe iron deficiency or mild iron deficiency affect bone. The protective or aggravating influence of different hormones (EPO, hepcidin, *etc.*) and factors such as inflammation, acidosis, and hypoxia due to anemia or other causes should be investigated. Moreover, genetic aspects of the interrelationship between iron and bone are largely unexplored.

Apart from the *in vitro* and animal studies, the main work should be done in human prospective studies. Hematologic and bone parameters should be determined during periods of several years and in different populations, children and adolescents, women at childbearing age and the elderly, with robust end points, such as diagnostic of bone disease and fracture.

## Abbreviations

ALP: Alkaline phosphatase
BMD: Bone mineral density
BMC: bone mineral content
BMI: body mass index
CTx: C-terminal cross-linked telopeptide of type I collagen
DPD: deoxypyridinoline
NTx: N-telopeptide cross-linked of type 1 collagen
25OHD: 25-hydroxyvitamin D
OC: osteocalcin
P1NP: N-terminal propeptide of type I procollagen
PYR: pyridinoline

## References

[1] Denic S., Agarwal M.M. (2007). Nutritional iron deficiency: An evolutionary perspective. Nutrition.

[2] Lieu P.T., Heiskala M., Peterson P.A., Yang Y. (2001). The roles of iron in health and disease. Mol. Asp. Med..

[3] Lynch S., Badham J., Zimmerman M.B., Kraemer K. (2007). Iron metabolism. The Guidebook Nutritional Anemia.

[4] Worldwide Prevalence of Anaemia 1993–2005. WHO Global Database on Anaemia. WHO Library Cataloguing-in-Publication Data: 2008. http://whqlibdoc.who.int/publications/2008/9789241596657_eng.pdf.

[5] Zimmermann M.B., Hurrell R.F. (2007). Nutritional iron deficiency. Lancet.

[6] McArthur J.O., Petocz P., Caterson I.D., Samman S. (2012). A randomized controlled trial in young women of the effects of consuming pork meat or iron supplements on nutritional status and feeling of well-being. J. Am. Coll. Nutr..

[7] Navas-Carretero S., Perez-Granados A.M., Sarria B., Carbajal A., Pedrosa M.M., Roe M.A., Fairweather-Tait S.J., Vaquero M.P. (2008). Oily fish increases iron bioavailability of a phytate rich meal in young iron deficient women. J. Am. Coll. Nutr..

[8] Navas-Carretero S., Perez-Granados A.M., Sarria B., Vaquero M.P. (2009). Iron absorption from meat pate fortified with ferric pyrophosphate in iron-deficient women. Nutrition.

[9] Hurrell R., Egli I. (2010). Iron bioavailability and dietary reference values. Am. J. Clin. Nutr..

[10] Vaquero M.P., Blanco-Rojo R., Toxqui L., Carbajal-Azcona A., Martínez-Roldán C. (2012). Nutrición y anemia. Manual Práctico de Nutrición y Salud.

[11] Toxqui L., Perez-Granados A.M., Blanco-Rojo R., Wright I., Gonzalez-Vizcayno C., Vaquero M.P. (2013). Effects of an iron or iron and vitamin d-fortified flavored skim milk on iron metabolism: A randomized controlled double-blind trial in iron-deficient women. J. Am. Coll. Nutr..

[12] Monsen E.R., Hallberg L., Layrisse M., Hegsted D.M., Cook J.D., Mertz W., Finch C.A. (1978). Estimation of available dietary iron. Am. J. Clin. Nutr..

[13] Blanco-Rojo R., Toxqui L., Lopez-Parra A.M., Baeza-Richer C., Perez-Granados A.M., Arroyo-Pardo E., Vaquero M.P. (2014). Influence of diet, menstruation and genetic factors on iron status: A cross-sectional study in Spanish women of childbearing age. Int. J. Mol. Sci..

[14] Toxqui L., Perez-Granados A.M., Blanco-Rojo R., Wright I., Vaquero M.P. (2014). A simple and feasible questionnaire to estimate menstrual blood loss: Relationship with hematological and gynecological parameters in young women. BMC Women’s Health.

[15] Harvey L.J., Armah C.N., Dainty J.R., Foxall R.J., John Lewis D., Langford N.J., Fairweather-Tait S.J. (2005). Impact of menstrual blood loss and diet on iron deficiency among women in the uk. Br. J. Nutr..

[16] Goddard A.F., McIntyre A.S., Scott B.B. (2000). Guidelines for the management of iron deficiency anaemia. British society of gastroenterology. Gut.

[17] Sarria B., Navas-Carretero S., Lopez-Parra A.M., Perez-Granados A.M., Arroyo-Pardo E., Roe M.A., Teucher B., Vaquero M.P., Fairweather-Tait S.J. (2007). The G277S transferrin mutation does not affect iron absorption in iron deficient women. Eur. J. Nutr..

[18] Benyamin B., McRae A.F., Zhu G., Gordon S., Henders A.K., Palotie A., Peltonen L., Martin N.G., Montgomery G.W., Whitfield J.B. (2009). Variants in TF and HFE explain approximately 40% of genetic variation in serum-transferrin levels. Am. J. Hum. Genet..

[19] Blanco-Rojo R., Baeza-Richer C., Lopez-Parra A.M., Perez-Granados A.M., Brichs A., Bertoncini S., Buil A., Arroyo-Pardo E., Soria J.M., Vaquero M.P. (2011). Four variants in transferrin and hfe genes as potential markers of iron deficiency anaemia risk: An association study in menstruating women. Nutr. Metab..

[20] Blanco-Rojo R., Bayele H.K., Srai S.K., Vaquero M.P. (2012). Intronic snp rs3811647 of the human transferrin gene modulates its expression in hepatoma cells. Nutr Hosp..

[21] Baeza-Richer C., Blanco-Rojo R., Lopez-Parra A.M., Brichs A., Bertoncini S., Perez-Granados A.M., Buil A., Soria J.M., Arroyo-Pardo E., Vaquero M.P. (2013). Identification of a novel quantitative trait nucleotype related to iron status in a calcium channel gene. Dis. Mark..

[22] Iron Deficiency Anaemia Assessment, Prevention, and Control. A Guide for Programme Managers; United Nations Children’s Fund; United Nations University; World Health Organization. http://www.who.int/nutrition/publications/micronutrients/anaemia_iron_deficiency/WHO_NHD_01.3/en/.

[23] Jauregui-Lobera I. (2014). Iron deficiency and cognitive functions. Neuropsychiatr. Dis. Treat..

[24] Jonker F.A., Boele van Hensbroek M. (2014). Anaemia, iron deficiency and susceptibility to infections. J. Infect..

[25] Buratti P., Gammella E., Rybinska I., Cairo G., Recalcati S. (2014). Recent advances in iron metabolism: Relevance for health, exercise, and performance. Med. Sci. Sports Exerc..

[26] Gibson R.S., Gibson R.S. (2005). Assesment of iron status. Principles of Nutritional Assessment.

[27] Abbaspour N., Hurrell R., Kelishadi R. (2014). Review on iron and its importance for human health. J. Res. Med. Sci..

[28] Toxqui L., de Piero A., Courtois V., Bastida S., Sanchez-Muniz F.J., Vaquero M.P. (2010). Iron deficiency and overload. Implications in oxidative stress and cardiovascular health. Nutr. Hosp..

[29] Ganz T. (2008). Iron homeostasis: Fitting the puzzle pieces together. Cell Metab..

[30] Ganz T. (2011). Hepcidin and iron regulation, 10 years later. Blood.

[31] Proff P., Romer P. (2009). The molecular mechanism behind bone remodelling: A review. Clin. Oral Investig..

[32] Manolagas S.C. (2000). Birth and death of bone cells: Basic regulatory mechanisms and implications for the pathogenesis and treatment of osteoporosis. Endocr. Rev..

[33] Boyce B.F., Rosenberg E., de Papp A.E., Duong le T. (2012). The osteoclast, bone remodelling and treatment of metabolic bone disease. Eur. J. Clin. Investig..

[34] Kular J., Tickner J., Chim S.M., Xu J. (2012). An overview of the regulation of bone remodelling at the cellular level. Clin. Biochem..

[35] Reynaga-Montecinos B., Zeni N. (2009). Biochemical markers of bone remodelling. Clinical utility. Acta Bioquím. Clín. Latinoam..

[36] Nakashima T., Hayashi M., Fukunaga T., Kurata K., Oh-Hora M., Feng J.Q., Bonewald L.F., Kodama T., Wutz A., Wagner E.F. (2011). Evidence for osteocyte regulation of bone homeostasis through RANKL expression. Nat. Med..

[37] Schaffler M.B., Cheung W.Y., Majeska R., Kennedy O. (2014). Osteocytes: Master orchestrators of bone. Calcif. Tissue Int..

[38] Bonewald L.F. (2011). The amazing osteocyte. J. Bone Miner. Res..

[39] Martin T.J., Sims N.A. (2015). RANKL/OPG; critical role in bone physiology. Rev. Endocr. Metab. Disord..

[40] Simonet W.S., Lacey D.L., Dunstan C.R., Kelley M., Chang M.S., Luthy R., Nguyen H.Q., Wooden S., Bennett L., Boone T. (1997). Osteoprotegerin: A novel secreted protein involved in the regulation of bone density. Cell.

[41] Boyce B.F., Xing L. (2008). Functions of RANKL/RANK/OPG in bone modeling and remodeling. Arch. Biochem. Biophys..

[42] Hadjidakis D.J., Androulakis II. (2006). Bone remodeling. Ann. N. Y. Acad. Sci..

[43] Matsuo K., Irie N. (2008). Osteoclast-osteoblast communication. Arch. Biochem. Biophys..

[44] Seeman E. (2009). Bone modeling and remodeling. Crit. Rev. Eukaryot. Gene Expr..

[45] Lorget F., Kamel S., Mentaverri R., Wattel A., Naassila M., Maamer M., Brazier M. (2000). High extracellular calcium concentrations directly stimulate osteoclast apoptosis. Biochem. Biophys. Res. Commun..

[46] Baim S., Miller P.D. (2009). Assessing the clinical utility of serum ctx in postmenopausal osteoporosis and its use in predicting risk of osteonecrosis of the jaw. J. Bone Min. Res..

[47] Shoulders M.D., Raines R.T. (2009). Collagen structure and stability. Ann. Rev. Biochem..

[48] Gorres K.L., Raines R.T. (2010). Prolyl 4-hydroxylase. Crit. Rev. Biochem. Mol. Biol..

[49] Tuderman L., Myllyla R., Kivirikko K.I. (1977). Mechanism of the prolyl hydroxylase reaction. 1. Role of co-substrates. Eur. J. Biochem..

[50] De Jong L., Kemp A. (1984). Stoicheiometry and kinetics of the prolyl 4-hydroxylase partial reaction. Biochim. Biophys. Acta.

[51] Pikuleva I.A., Waterman M.R. (2013). Cytochromes P450: Roles in diseases. J. Biol. Chem..

[52] Sakaki T., Kagawa N., Yamamoto K., Inouye K. (2005). Metabolism of vitamin D3 by cytochromes P450. Front. Biosci..

[53] Dong Q., Miller W.L. (2004). Vitamin D 25-hydroxylase deficiency. Mol. Genet. Metab..

[54] Holick M.F. (2009). Vitamin D status: Measurement, interpretation, and clinical application. Ann. Epidemiol..

[55] Holick M.F., Chen T.C. (2008). Vitamin D deficiency: A worldwide problem with health consequences. Am. J. Clin. Nutr..

[56] Fu G.K., Lin D., Zhang M.Y., Bikle D.D., Shackleton C.H., Miller W.L., Portale A.A. (1997). Cloning of human 25-hydroxyvitamin D-1 alpha-hydroxylase and mutations causing vitamin d-dependent rickets type 1. Mol. Endocrinol..

[57] Schlingmann K.P., Kaufmann M., Weber S., Irwin A., Goos C., John U., Misselwitz J., Klaus G., Kuwertz-Broking E., Fehrenbach H. (2011). Mutations in CYP24A1 and idiopathic infantile hypercalcemia. N. Engl. J. Med..

[58] Jones G., Prosser D.E., Kaufmann M. (2014). Cytochrome P450-mediated metabolism of vitamin D. J. Lipid Res..

[59] Vogiatzi M.G., Macklin E.A., Fung E.B., Cheung A.M., Vichinsky E., Olivieri N., Kirby M., Kwiatkowski J.L., Cunningham M., Holm I.A. (2009). Bone disease in thalassemia: A frequent and still unresolved problem. J. Bone Miner. Res..

[60] Weinberg E.D. (2008). Role of iron in osteoporosis. Pediatr. Endocrinol. Rev..

[61] Guggenbuhl P., Deugnier Y., Boisdet J.F., Rolland Y., Perdriger A., Pawlotsky Y., Chales G. (2005). Bone mineral density in men with genetic hemochromatosis and HFE gene mutation. Osteoporos. Int..

[62] Lee K.S., Jang J.S., Lee D.R., Kim Y.H., Nam G.E., Han B.D., Do Han K., Cho K.H., Kim S.M., Choi Y.S. (2014). Serum ferritin levels are positively associated with bone mineral density in elderly Korean men: The 2008–2010 Korea National Health and Nutrition Examination Surveys. J. Bone Miner. Metab..

[63] Kim B.J., Ahn S.H., Bae S.J., Kim E.H., Lee S.H., Kim H.K., Choe J.W., Koh J.M., Kim G.S. (2012). Iron overload accelerates bone loss in healthy postmenopausal women and middle-aged men: A 3-year retrospective longitudinal study. J. Bone Miner. Res..

[64] Guggenbuhl P., Brissot P., Loreal O. (2011). Miscellaneous non-inflammatory musculoskeletal conditions. Haemochromatosis: The bone and the joint. Best Pract. Res. Clin. Rheumatol..

[65] Buyukbese M.A., Cetinus E., Cetinkaya A., Aras S. (2005). Ferritin levels in postmenopausal women do not seem to play a significant role in osteoporosis. South. Med. J..

[66] Terpos E., Voskaridou E. (2010). Treatment options for thalassemia patients with osteoporosis. Ann. N. Y. Acad. Sci..

[67] De Sanctis V., Soliman A.T., Elsedfy H., Yassin M., Canatan D., Kilinc Y., Sobti P., Skordis N., Karimi M., Raiola G. (2013). Osteoporosis in thalassemia major: An update and the I-CET 2013 recommendations for surveillance and treatment. Pediatr. Endocrinol. Rev..

[68] Voskaridou E., Terpos E. (2004). New insights into the pathophysiology and management of ossteoporosis in patients with beta thalasaemia. Br. J. Haematol..

[69] Nakchbandi I.A. (2014). Osteoporosis and fractures in liver disease: Relevance, pathogenesis and therapeutic implications. World J. Gastroenterol..

[70] Miller R.G., Segal J.B., Ashar B.H., Leung S., Ahmed S., Siddique S., Rice T., Lanzkron S. (2006). High prevalence and correlates of low bone mineral density in young adults with sickle cell disease. Am. J. Hematol..

[71] Gupta R., Marouf R., Adekile A. (2010). Pattern of bone mineral density in sickle cell disease patients with the high-Hb F phenotype. Acta Haematol..

[72] Sarrai M., Duroseau H., D’Augustine J., Moktan S., Bellevue R. (2007). Bone mass density in adults with sickle cell disease. Br. J. Haematol..

[73] Reynolds J. (1966). A re-evaluation of the “fish vertebra” sign in sickle cell hemoglobinopathy. Am. J. Roentgenol. Radium Ther. Nucl. Med..

[74] Voskaridou E., Stoupa E., Antoniadou L., Premetis E., Konstantopoulos K., Papassotiriou I., Terpos E. (2006). Osteoporosis and osteosclerosis in sickle cell/beta-thalassemia: The role of the rankl/osteoprotegerin axis. Haematologica.

[75] Valenti L., Varenna M., Fracanzani A.L., Rossi V., Fargion S., Sinigaglia L. (2009). Association between iron overload and osteoporosis in patients with hereditary hemochromatosis. Osteoporos. Int..

[76] Ebrahimpour L., Akhlaghpoor S., Azarkayvan A., Salehi M., Morteza A., Alinaghi R. (2012). Correlation between bone mineral densitometry and liver/heart iron overload evaluated by quantitative T2* MRI. Hematology.

[77] Vogiatzi M.G., Macklin E.A., Fung E.B., Vichinsky E., Olivieri N., Kwiatkowski J., Cohen A., Neufeld E., Giardina P.J. (2006). Prevalence of fractures among the thalassemia syndromes in North America. Bone.

[78] Chan Y.L., Pang L.M., Chik K.W., Cheng J.C., Li C.K. (2002). Patterns of bone diseases in transfusion-dependent homozygous thalassaemia major: Predominance of osteoporosis and desferrioxamine-induced bone dysplasia. Pediatr. Radiol..

[79] Vogiatzi M.G., Autio K.A., Schneider R., Giardina P.J. (2004). Low bone mass in prepubertal children with thalassemia major: Insights into the pathogenesis of low bone mass in thalassemia. J. Pediatr. Endocrinol. Metab..

[80] Shah F.T., Chatterjee R., Owusu-Asante M., Porter J.B. (2004). Adults with severe sickle cell anaemia and iron overload have a high incidence of osteopenia and osteoporosis. Blood..

[81] Tsay J., Yang Z., Ross F.P., Cunningham-Rundles S., Lin H., Coleman R., Mayer-Kuckuk P., Doty S.B., Grady R.W., Giardina P.J. (2010). Bone loss caused by iron overload in a murine model: Importance of oxidative stress. Blood.

[82] Mahachoklertwattana P., Sirikulchayanonta V., Chuansumrit A., Karnsombat P., Choubtum L., Sriphrapradang A., Domrongkitchaiporn S., Sirisriro R., Rajatanavin R. (2003). Bone histomorphometry in children and adolescents with beta-thalassemia disease: Iron-associated focal osteomalacia. J. Clin. Endocrinol. Metab..

[83] Medeiros D.M., Stoecker B., Plattner A., Jennings D., Haub M. (2004). Iron deficiency negatively affects vertebrae and femurs of rats independently of energy intake and body weight. J. Nutr..

[84] Katsumata S., Tsuboi R., Uehara M., Suzuki K. (2006). Dietary iron deficiency decreases serum osteocalcin concentration and bone mineral density in rats. Biosci. Biotechnol. Biochem..

[85] Katsumata S., Katsumata-Tsuboi R., Uehara M., Suzuki K. (2009). Severe iron deficiency decreases both bone formation and bone resorption in rats. J. Nutr..

[86] Diaz-Castro J., Lopez-Frias M.R., Campos M.S., Lopez-Frias M., Alferez M.J., Nestares T., Ojeda M.L., Lopez-Aliaga I. (2012). Severe nutritional iron-deficiency anaemia has a negative effect on some bone turnover biomarkers in rats. Eur. J. Nutr..

[87] Harris M.M., Houtkooper L.B., Stanford V.A., Parkhill C., Weber J.L., Flint-Wagner H., Weiss L., Going S.B., Lohman T.G. (2003). Dietary iron is associated with bone mineral density in healthy postmenopausal women. J. Nutr..

[88] Maurer J., Harris M.M., Stanford V.A., Lohman T.G., Cussler E., Going S.B., Houtkooper L.B. (2005). Dietary iron positively influences bone mineral density in postmenopausal women on hormone replacement therapy. J. Nutr..

[89] D’Amelio P., Cristofaro M.A., Tamone C., Morra E., Di Bella S., Isaia G., Grimaldi A., Gennero L., Gariboldi A., Ponzetto A. (2008). Role of iron metabolism and oxidative damage in postmenopausal bone loss. Bone.

[90] Blanco-Rojo R., Perez-Granados A.M., Toxqui L., Zazo P., de la Piedra C., Vaquero M.P. (2013). Relationship between vitamin d deficiency, bone remodelling and iron status in iron-deficient young women consuming an iron-fortified food. Eur. J. Nutr..

[91] Toxqui L., Perez-Granados A.M., Blanco-Rojo R., Wright I., de la Piedra C., Vaquero M.P. (2014). Low iron status as a factor of increased bone resorption and effects of an iron and vitamin D-fortified skimmed milk on bone remodelling in young spanish women. Eur. J. Nutr..

[92] Zhao G.Y., Zhao L.P., He Y.F., Li G.F., Gao C., Li K., Xu Y.J. (2012). A comparison of the biological activities of human osteoblast hFOB1.19 between iron excess and iron deficiency. Biol. Trace Elem. Res..

[93] Díaz-Castro J., Ramirez Lopez-Frias M., Campos M.S., Lopez-Frias M., Alferez M.J., Nestares T., Ortega E., Lopez-Aliaga I. (2011). Goat milk during iron repletion improves bone turnover impaired by severe iron deficiency. J. Dairy Sci..

[94] Kim B.J., Lee S.H., Koh J.M., Kim G.S. (2013). The association between higher serum ferritin level and lower bone mineral density is prominent in women ≥45 years of age (KNHANES 2008–2010). Osteoporos. Int..

[95] Wright I., Blanco-Rojo R., Fernandez M.C., Toxqui L., Moreno G., Perez-Granados A.M., de la Piedra C., Remacha A.F., Vaquero M.P. (2013). Bone remodelling is reduced by recovery from iron-deficiency anaemia in premenopausal women. J. Physiol. Biochem..

[96] Blanco-Rojo R., Perez-Granados A.M., Toxqui L., Gonzalez-Vizcayno C., Delgado M.A., Vaquero M.P. (2011). Efficacy of a microencapsulated iron pyrophosphate-fortified fruit juice: A randomised, double-blind, placebo-controlled study in Spanish iron-deficient women. Br. J. Nutr..

[97] Arnett T.R., Gibbons D.C., Utting J.C., Orriss I.R., Hoebertz A., Rosendaal M., Meghji S. (2003). Hypoxia is a major stimulator of osteoclast formation and bone resorption. J. Cell. Physiol..

[98] Shiozawa Y., Jung Y., Ziegler A.M., Pedersen E.A., Wang J., Wang Z., Song J., Wang J., Lee C.H., Sud S. (2010). Erythropoietin couples hematopoiesis with bone formation. PLoS ONE.

[99] Ilich-Ernst J.Z., McKenna A.A., Badenhop N.E., Clairmont A.C., Andon M.B., Nahhas R.W., Goel P., Matkovic V. (1998). Iron status, menarche, and calcium supplementation in adolescent girls. Am. J. Clin. Nutr..

[100] Hiram-Bab S., Liron T., Deshet-Unger N., Mittelman M., Gassmann M., Rauner M., Franke K., Wielockx B., Neumann D., Gabet Y. (2015). Erythropoietin directly stimulates osteoclast precursors and induces bone loss. FASEB J..

[101] Lee M.Y., Fukunaga R., Lee T.J., Lottsfeldt J.L., Nagata S. (1991). Bone modulation in sustained hematopoietic stimulation in mice. Blood.

[102] Okito A., Nakahama K.I., Akiyama M., Ono T., Morita I. (2015). Involvement of the G-protein-coupled receptor 4 in RANKL expression by osteoblasts in an acidic environment. Biochem. Biophys. Res. Commun..

